# Dosimetric Feasibility of Dose-Painting Radiation Therapy for Targeting Hypoxia in Prostate Cancer on a Novel Ring Gantry Radiation Therapy System

**DOI:** 10.1016/j.adro.2025.101940

**Published:** 2025-11-29

**Authors:** Tanguy Perennec, Karine A. Al Feghali, Dorine de Jong, Oluwaseyi M. Oderinde, Grant Gibbard, Mélanie Dore, Gregory Delpon, Moignier Alexandra, Yves Seroux, Ludovic Ferrer, Matthieu Hatt, Caroline Rousseau, Stéphane Supiot

**Affiliations:** a Department of Radiation Oncology, Institut de Cancérologie de l’Ouest, Nantes, St-Herblain, France; b RefleXion Medical Inc., Hayward, California; c Advanced Molecular Imaging in Radiotherapy (AdMIRe) Research Laboratory, Purdue University, West Lafayette, Indiana; d Centre de Recherche en Cancérologie Immunologie Nantes Angers (CRCINA), Institut National de Santé et de la Recherche Médicale (INSERM) UMR U1232, Centre National de la Recherche Scientifique (CNRS) ERL 6001, Université de Nantes, Nantes, France; e Department of Nuclear Medicine, Institut de Cancérologie de l’Ouest, Nantes, St-Herblain, France; f Laboratoire de Traitement de l’Information Médicale (LATIM), INSERM, UMR 1101, Université de Bretagne Occidentale, IBSAM, faculté de médecine, Brest CEDEX, France; g Laboratoire US2B, UMR CNRS 6286, Nantes, France

## Abstract

**Purpose:**

Hypoxia is a well-known major factor contributing to the radioresistance of prostate cancer, which could be counteracted by increasing the dose. This study aimed to demonstrate the dosimetric feasibility of a dose-painting radiation therapy plan for prostate cancer, using a novel ring gantry system, based on the localization of tumoral and hypoxic areas.

**Methods and Materials:**

Seven patients from the Programme d’Action Intégré de Recherche-prostate study, who underwent external-beam radiation therapy for intermediate-risk prostate cancer and exhibited pretherapeutic fluromisonodazole positron emission tomography (PET) uptake in the tumor, were selected. The gross tumor volume (GTV) was delineated on the magnetic resonance imaging, and the hypoxic region within the planning target volume was delineated based on fluromisonodazole PET uptake. Intensity modulated radiation therapy planning was performed based on 3 different prescriptions: standard fractionation (77 Gy in 35 fractions to the planning target volume), with an integrated boost of 95 Gy and 118 Gy in 35 fractions to the GTV and the hypoxic region, moderate hypofractionation (60 Gy in 20 fractions) with a boost of 67 Gy and 91 Gy to the GTV and the hypoxic region, and high hypofractionation (40 Gy in 5 fractions) with a boost of 50 Gy to the GTV and as high as possible to the hypoxic region. Planning was performed on the research version of the RefleXion treatment planning system.

**Results:**

We achieved the prescribed dose in all 7 patients while respecting the usual dose limits for organs at risk.

**Conclusions:**

This study demonstrated the dosimetric feasibility of dose escalation in both the tumor and hypoxic regions in patients with prostate cancer using the RefleXion treatment planning system, without compromising the dose limits for organs at risks.

## Introduction

Several trials have successfully shown that dose escalation in prostate cancer can improve recurrence-free survival.^1^, ^2^, ^3^, ^4^, ^5^ Retrospective data on small samples suggest that local recurrences after radiation therapy occur more than 80% of the time in the index zone,^6^, ^7^, ^8^ encouraging localized dose escalation to tumoral volume. Some studies already investigated the feasibility of such a strategy, with conventionally fractionated radiation therapy,^9^ moderate hypofractionated radiation therapy,^10^^,^^11^ and high-hypofractionated radiation therapy.^12^

Tumor hypoxia is a major radioresistance factor, inhibiting the production of reactive oxygen species during irradiation and selecting radioresistant cells.^13^ In the specific case of prostate cancer, it has been shown that direct invasive measurement of oxygen levels or expression of hypoxia-related genes measured by biopsy can predict the aggressiveness and recurrence of the tumor after irradiation.^14^^,^^15^ Therefore, we could consider dose escalation in the hypoxic volume and the dose escalation in the gross tumor volume (GTV), using a dose-painting strategy. This approach would make it possible to increase the dose in zones of interest while limiting the dose to organs at risk (OAR), compared with increasing the dose in the entire prostate. Delineation of the target zone would require accurate mapping of these volumes by functional imaging. In contrast, biopsies would not be able to accurately reflect the spatial extent of the hypoxia. Fluromisonodazole (F-MISO) is known to bind hypoxic cells^16^ and can detect hypoxic regions in approximately one-third of intermediate-risk prostate cancer patients undergoing radiation therapy.^17^

The RefleXion X1 ring-gantry radiation therapy system integrates a linear accelerator with dual-modality imaging (kilo Voltage Computed Tomography [kVCT] and positron emission tomography [PET]) to enable biology-guided radiation therapy (BgRT), whereby real-time PET emissions from the tumor steer subsecond radiation beamlets to the target.^18^ By creating a feedback loop between real-time PET functional images as a surrogate for tumor biology and radiation delivery, the X1 system can dynamically adapt treatment as the tumor moves.

In this study, we aimed to demonstrate the feasibility of tumoral and hypoxia-guided dose-escalated radiation therapy in prostate cancer using the research version of the RefleXion treatment planning system (X1-TPS), with conventionally fractionated and hypofractionated regimens.

## Patients and Methods

### Patient selection

The local ethics committee approved this prospective study. Written consent was obtained from all participants. Inclusion criteria were NCCN-defined intermediate-risk prostate cancer patients (Gleason 6, PSA 10-20 ng/mL; or Gleason 7, PSA <20 ng/mL; *T* < T3) in whom high-dose radiation therapy to the prostate was indicated. We excluded patients receiving, or having received, hormonal treatment.

### Magnetic resonance imaging

All patients underwent a trans-pelvic coil 1.5 T magnetic resonance imaging (MRI) (Aera 1.5T, Siemens Healthineers) in the supine position with Gadolinium injection using a surface coil before radiation therapy. Axial T2-weighted imaging was performed. Images were reconstructed in a voxel dimension of 0.31 × 0.31 × 4 mm^3^. Isotropic axial diffusion-weighted scans were performed using a single-shot echo-planar imaging sequence.

### Fluromisonodazole PET/CT

All patients underwent an F-MISO (IASON Gmbh) PET/CT using a Siemens Biograph mCT40 before radiation therapy. Acquisition started immediately after intravenous injection gathering dynamic PET images on the pelvis over 45 minutes on list mode. Pelvic PET images used for planning radiation therapy were acquired 3.5 hours after injection, with an acquisition time of 10 minutes; we did not use dynamic data. Low-dose CT in the supine position was performed for localization and attenuation correction. Regions of interest were drawn over the *Gluteus Minimus* muscle and the hottest areas of F-MISO uptake. A tumor-to-muscle-ratio threshold of 1.4 was chosen to identify the high F-MISO uptake regions, similar to other published studies.^19^^,^^20^ All uptakes in F-MISO were semi-automatically delineated using the fuzzy locally adaptive Bayesian algorithm, developed specifically for PET image segmentation.^21^^,^^22^

### Planning CT and image coregistration

Patients were asked to empty the rectum and bladder and then drink 500 mL of water 60 minutes before the planned CT scan (BigBore, Philips). Patients were scanned (3-mm-thick slices every 3 mm) in the supine position with conventional head and knee support.

Image coregistration of F-MISO, MRI, and planning CT images was performed using a rigid, nonparametric, affine transformation (IPlan RTimage 4.1). To account for bladder and rectal filling, automatic fusion was adjusted manually based on outer prostate volume and intraprostatic calcifications.

### Volume definition

GTVhypoxic was defined by the hypoxic volume detected on F-MISO PET/CT before radiation therapy, semi-automatically delineated as described in the F-MISO PET/CT section.

Gross tumor volume was defined as a macroscopic tumor, detected on the T2W MRI and manually contoured by 2 radiation oncologists using RayStation software (RaySearchLabs).

Three clinical target volumes (CTVs) were defined as follows:

CTV1: The entire prostate, assessed with the help of the MRI for the inferior limit.
CTV2: Volume of CTV1 containing GTV with an isotropic margin of 0.5 cm.
BTV (biological target volume): GTVhypoxic with an isotropic margin of 0.5 cm.

**Figure 1:**
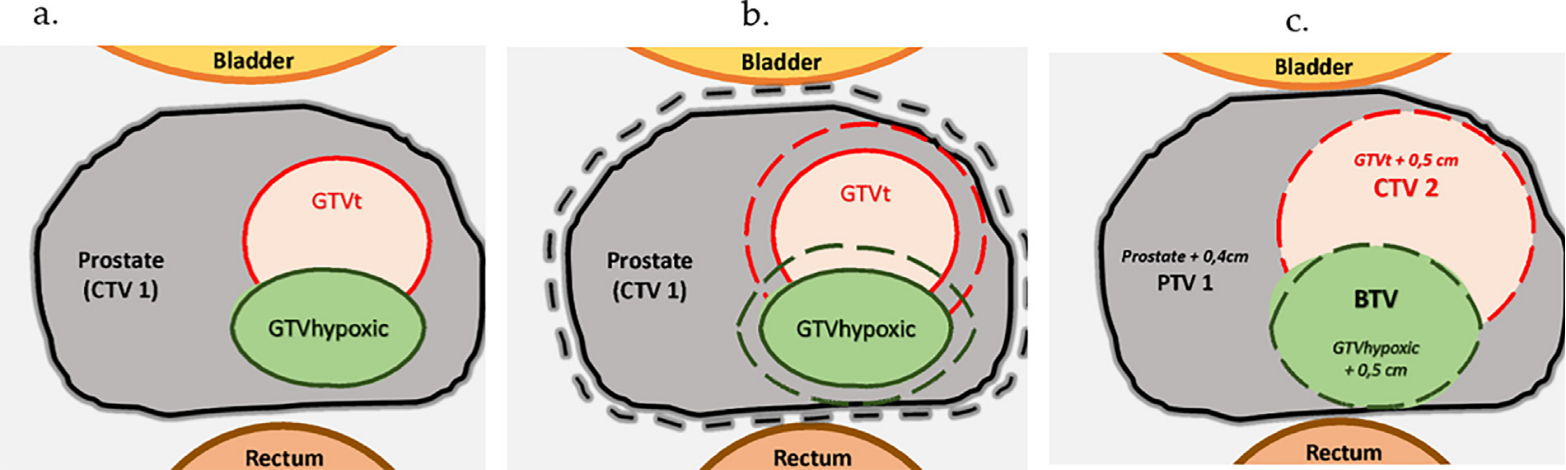
Representation of the target volumes gross tumoral volumes defined on magnetic resonance imaging (MRI) (target gross tumor volume [GTVt]) and on fluromisonodazole (F-MISO) (GTVhypoxic) (a), with an isotropic margin of 0.5 cm for the GTVt and GTVhypoxic and 0.4 cm for the whole prostate (clinical target volume [CTV1]) (b) to define the CTV2, biological target volume (BTV), and planned target volume (PTV1), respectively (c).

The planned target volume (PTV) was defined as CTV1 with an isotropic margin of 0.4 cm, excluding the rectum and the bladder. A schematic representation of the target volumes is provided in Fig. 1.

OAR include the rectum (from the anorectal junction to the recto-sigmoidal junction), the bladder, the urethra, and the femoral heads.

The target volumes were defined for the 7 patients as shown in Fig. 1.

### Treatment planning

Planning was performed on the research version of the X1-TPS (RefleXion). Dose statistics for the PTV, GTV, and BTV were calculated on the entire volume of each structure, without subtracting overlapping regions.

For the conventionally fractionated plan, we planned a mean dose of 77 Gy in 35 fractions of 2.2 Gy within the planning target volume (PTV1) with a boost of 95 Gy in 35 fractions of 2.7 Gy to the macroscopic tumoral volume (CTV2) as prescribed in the FLAME-Trial.^9^ Then, a median dose of 95 Gy to the BTV was prescribed in all patients. Finally, we increased the dose prescribed to the BTV to 118 Gy or until a dose constraint was reached. The OAR constraints were like the FLAME-Trial: rectum: V77 Gy < 1 cc, V72 Gy < 5%, V50 Gy < 50%; bladder: V80 Gy < 1 cc, V72 Gy < 10%; and femoral heads: V50 Gy < 5%.

For the moderate hypofractionated plan, based on the DELINEATE trial,^10^ we planned a dose of 60 Gy in 20 fractions of 3 Gy to the whole prostate and a boost of 67 Gy in 20 fractions of 3.35 Gy to the CTV2, as well as a dose up to 91 Gy to the BTV. Dose constraints were as follows: rectum, V40.5 < 60%; V48.7 < 50%; V52.7 < 30 %; V56.8 < 15 %; V60.8 < 5%; and D2% < 76 Gy; bladder, V52.7 < 50%; V56.8 < 35%; V60.8 < 25%; and V64.9 < 15%; and femoral heads, V40.5 < 50%.

For the high-hypofractionated plan, based on a previously published dose-escalated stereotactic ablative radiation therapy plan, we created a plan of 40 Gy in 5 fractions of 8 Gy,^12^ and we increased the dose prescribed to the BTV until a dose constraint was met dose constraints were as follows: rectum, V28Gy < 15%; V35Gy < 5%; and V50Gy < 50%; bladder, V28Gy < 15%.

## Results

### Patient population

We recruited 7 patients with histologically proven intermediate-risk prostate cancer, treated from September 2012 to October 2014 in the Programme d’Action Intégré de Recherche-prostate study. They all had F-MISO PET/CT uptake before radiation therapy.

**Figure 2:**
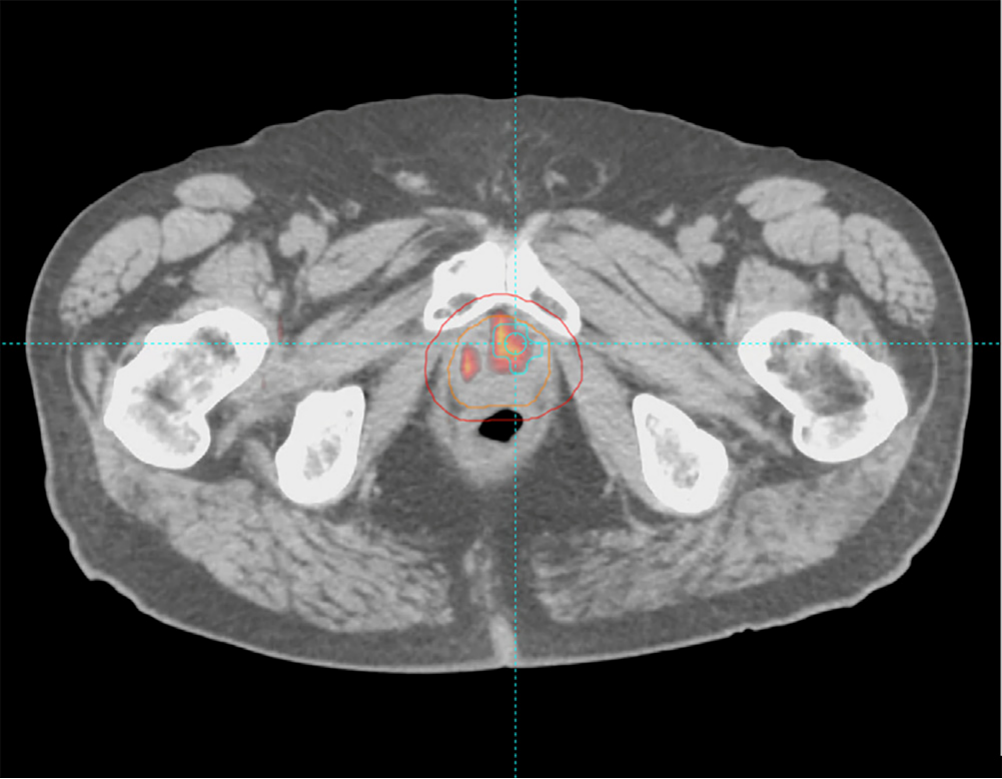
Hypoxic biological target volume (BTV), delineated in Cyan on fluromisonodazole (F-MISO) positron emission tomography (PET), clinical target volume (CTV1) prostate in orange, and planned target volume (PTV1) in red.

The average size of the GTV and BTV was 11.4cc (SD, 11.3) and 1.30cc (SD, 1.20), respectively. An example of a delineated BTV is provided in Fig. 2.

### Radiation therapy planning

For the standard fractionation regimen, the average dose to the PTV was 71.3 ± 11.7 Gy for D98% and 105.0 ± 7.6 Gy for D2%. The GTV received an average dose of 90.3 ± 6.9 Gy for D98% and 113.2 ± 6.9 Gy for D2%, whereas the BTV had an average D98% of 107.4 ± 5.6 Gy and D2% of 116.0 ± 5.6 Gy.

Using the moderate hypofractionation technique, the average D98% to the PTV was 54.3 ± 2.5 Gy, and the D2% was 83.6 ± 7.0 Gy. For the GTV, the D98% was 61.2 ± 6.2 Gy, and the D2% was 89.8 ± 5.9 Gy, whereas the BTV received an average D98% of 85.2 ± 4.1 Gy and D2% of 92.3 ± 3.2 Gy.

With the high hypofractionation technique, the average D98% to the PTV was 31.7 ± 5.3 Gy, and the D2% was 55.8 ± 2.9 Gy. The GTV received an average D98% of 45.7 ± 4.9 Gy and D2% of 59.2 ± 3.2 Gy, whereas the BTV had an average D98% of 53.6 ± 4.2 Gy and D2% of 60.6 ± 2.5 Gy.

**Figure 3:**
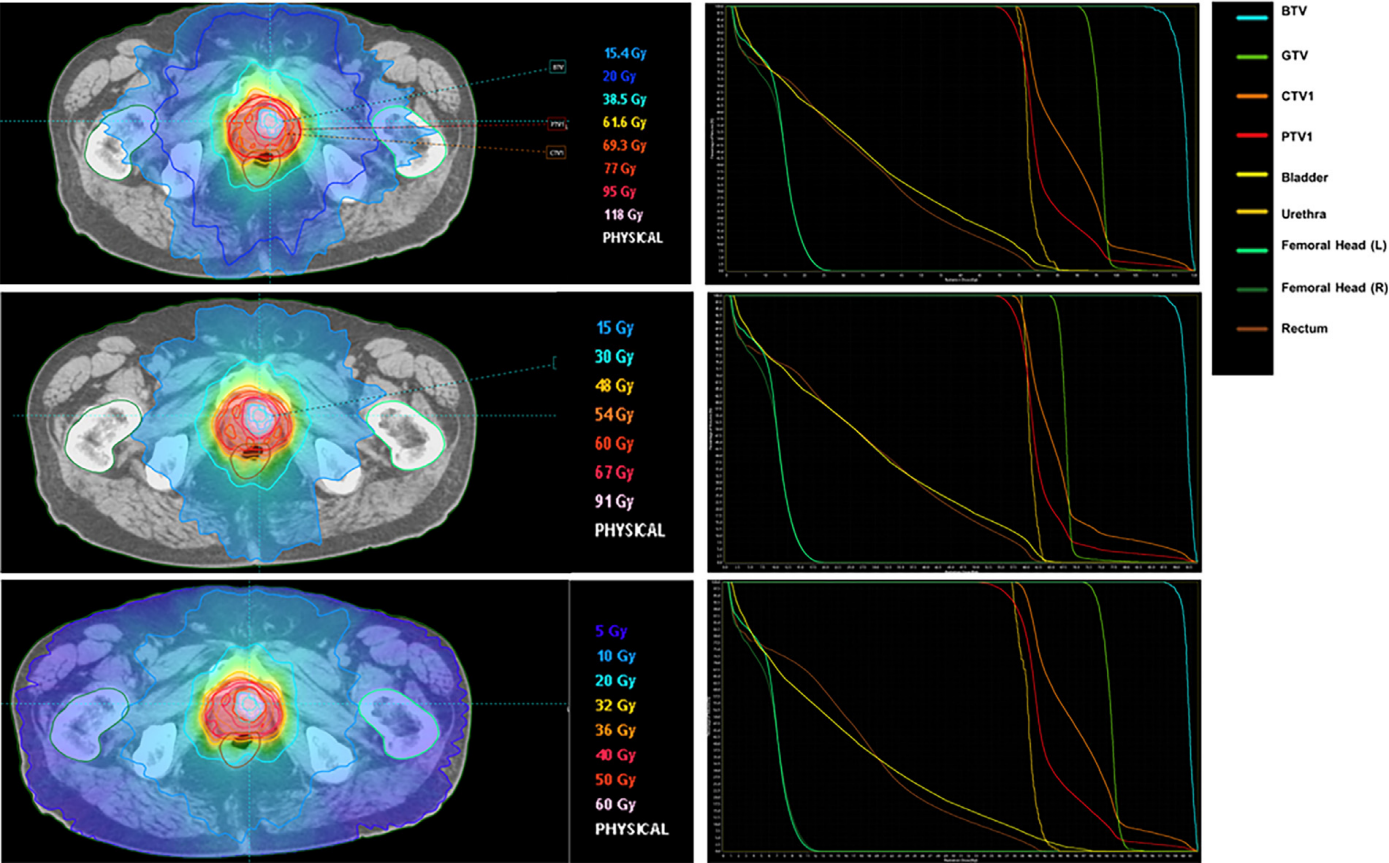
Examples of intensity modulated radiation therapy (IMRT) standard fractionation, moderate, and high hypofractionation plans for the same prostate cancer patient (left column) with respective computed plan dose-volume histograms (right column).

An example of each dosimetric plan for 1 of the patients is shown in Fig. 3.

## Discussion

We hypothesized that a dosimetric plan with a simultaneous integrated boost (SIB) to the hypoxic volume was feasible while respecting the same constraints to the organs at risk as previous trials evaluating the feasibility of SIB on the tumoral volume only have demonstrated. We increased the dose in the hypoxic subregion in all patients, without reaching the constraints of the surrounding organ at risk.

The RefleXion X1 is a radiation therapy delivery system that could enable the clinical implementation of such an approach, allowing for biology-guided radiation therapy with high spatial precision. Quality assurance procedures were performed and showed excellent agreement between measured and planned values, supporting the technical feasibility and accuracy of this delivery method.^23^

Hypoxic radioresistance is given by the hypoxia reduction factor (HRF), ie, the ratio of the doses for a specific effect under 2 different oxygenation conditions. HRF at hypoxic levels is 3 but may be inferior with hypofractionation and reoxygenation during irradiation.^24^ This means that the total dose needs to be increased by a factor of 3 to achieve the same cell killing in hypoxic areas.^25^ α/β in prostate cancer is known to be low, approximately 1.5 to 3, whereas it is higher in the rectum and much higher in the bladder.^26^ Hypofractionation will lead to a substantial increase in the equivalent dose, whereas the organs at risk will be slightly impacted, favoring the use of high-hypofractionated radiation therapy.

It should be noted that some conditions must be met in practice to apply these plans: patients should be able to have an MRI and not have metallic implants, such as hip prostheses, that could cause significant artifacts. In addition, reaching very high doses in some volumes requires that these volumes are not contiguous to an organ at risk.

We previously showed that reoxygenation occurred during conventionally fractionated radiation therapy in all patients.^27^ After a minimum dose of 20 Gy, hypoxic volumes decreased or were no longer detected in 4 and 5 of 9 patients. This suggests that the hypoxic subvolumes could be adjusted to the reduction of the hypoxic volume during radiation therapy or that Stereotactic Body Radiation Therapy (SBRT) to the hypoxic region before conventionally fractionated radiation therapy represents an interesting option, rather than an SIB.^28^ In a similar feasibility study, an SIB to hypoxic volume based on FAZA PET/CT in patients with head and neck cancers reached a median dose of 86 Gy within the hypoxic volume.^29^ In this study, 3 FAZA PET/CTs were done during irradiation to adjust dosimetry according to reoxygenation during treatment.

Our paper presents some limitations. We decided to prescribe a homogeneous dose to the hypoxic tumoral volume rather than a graded dose based on SUV uptake in the F-MISO PET/CT because a correlation between the intensity of hypoxia and SUV uptake in humans has not been demonstrated. However, a dose-painting strategy based on tumor control probability per voxel, calculated with MRI, CT, F-MISO PET/CT, PSMA, and choline PET/CT, is feasible and could be an interesting approach.^30^

In this study, all FMISO-positive hypoxic subvolumes were included in the boost volume, irrespective of their overlap with the MRI-defined GTV. Although some hypoxic regions may lie outside histologically confirmed tumor areas, hypoxia has been associated with tumor aggressiveness and radioresistance. Moreover, given the limited spatial resolution of PET imaging (approximately 4 mm), small misregistrations or partial volume effects may result in apparent hypoxic areas outside tumor boundaries. Including the full FMISO-positive volume therefore enabled an assessment of the feasibility of encompassing all potentially relevant hypoxic regions. However, biological validation is required to determine whether boosting hypoxia beyond the tumor truly improves outcomes.

The dose constraints used correspond to those in the respective published trials. Note that some standard dose constraints, such as the 50% isodose covering the entire circumference of the rectum, were not included in the protocol and therefore were not attempted.

Patients in this study did not undergo fiducial marker implantation, and image guidance relied on natural calcifications. In future dose-escalation settings, implanted fiducials could enhance targeting accuracy. In addition, precise identification and tracking of small hypoxic regions during treatment, which can move with changes in bladder or rectal filling, remain challenging. Besides adding a margin, the integration of PET imaging on the treatment machine could help overcome this limitation by enabling real-time, anatomically accurate targeting of hypoxic subvolumes. Another limitation of this study is the small proportion of patients with hypoxic PET volumes in the study population: only 9 of 27 patients (33%) with intermediate-risk prostate cancer had an SUV uptake on F-MISO PET/CT. However, patients with hypoxic volumes are known to be more at risk of recurrence^14^^,^^15^ and are the most likely to benefit from a dose increase. The dose-painting strategy is a personalized treatment that is not meant to be generalized to all patients. In addition, 2 patients out of 9 had a hypoxic volume on PET scan, but were not able to have an MRI or had bilateral hip prostheses, which is a technical limitation to the generalization of this strategy.

Finally, although the concept of dose escalation to hypoxic subvolumes has already been investigated in clinical trials for head and neck^31^ and lung cancers,^32^ this strategy has not yet been evaluated in prostate cancer. Our study represents an initial dosimetric step in this direction. Given that BgRT is currently only cleared by the US Food and Drug Administration for the treatment of lung and bone tumors using 18F-FDG. F-MISO is not cleared as a BgRT bioguide and is not used to treat patients on the X1 machine. The next logical step would be the development of prospective clinical trials to assess the clinical benefit and feasibility of hypoxia-guided dose escalation in prostate cancer.

## Conclusion

It is possible to reach very high doses in specific regions of the prostate in patients with macroscopic tumoral volume and hypoxic volume, regardless of the fractionation. This strategy may only be considered in selected patients, with an F-MISO PET uptake noncontiguous to organs at risk and an MRI without artifact.

## Disclosures

Stéphane Supiot reports that financial support was provided by RefleXion Medical and INCA PAIR prostate. The other authors declare that they have no known competing financial interests or personal relationships that could have appeared to influence the work reported in this paper.
